# L-DOPA and oxytocin influence the neural correlates of performance monitoring for self and others

**DOI:** 10.1007/s00213-024-06541-9

**Published:** 2024-01-29

**Authors:** Myrthe Jansen, Sandy Overgaauw, Ellen R. A. de Bruijn

**Affiliations:** 1 Department of Clinical Psychology, Institute of Psychology, Leiden University, Leiden, The Netherlands; 2 Leiden Institute for Brain and Cognition (LIBC), Leiden, The Netherlands

**Keywords:** Social performance monitoring, Dopamine, Oxytocin, Functional magnetic resonance imaging (fMRI), Prosocial

## Abstract

**Rationale:**

The ability to monitor the consequences of our actions for others is imperative for flexible and adaptive behavior, and allows us to act in a (pro)social manner. Yet, little is known about the neurochemical mechanisms underlying alterations in (pro)social performance monitoring.

**Objective:**

The aim of this functional magnetic resonance imaging (fMRI) study was to improve our understanding of the role of dopamine and oxytocin and their potential overlap in the neural mechanisms underlying performance monitoring for own versus others’ outcomes.

**Method:**

Using a double-blind placebo-controlled cross-over design, 30 healthy male volunteers were administered oxytocin (24 international units), the dopamine precursor L-DOPA (100 mg + 25 mg carbidopa), or placebo in three sessions. Participants performed a computerized cannon shooting game in two recipient conditions where mistakes resulted in negative monetary consequences for (1) oneself or (2) an anonymous other participant.

**Results:**

Results indicated reduced error-correct differentiation in the ventral striatum after L-DOPA compared to placebo, independent of recipient. Hence, pharmacological manipulation of dopamine via L-DOPA modulated performance-monitoring activity in a brain region associated with reward prediction and processing in a domain-general manner. In contrast, oxytocin modulated the BOLD response in a recipient-specific manner, such that it specifically enhanced activity for errors that affected the other in the pregenual anterior cingulate cortex (pgACC), a region previously implicated in the processing of social rewards and prediction errors. Behaviorally, we also found reduced target sizes—indicative of better performance—after oxytocin, regardless of recipient. Moreover, after oxytocin lower target sizes specifically predicted higher pgACC activity when performing for others.

**Conclusions:**

These different behavioral and neural patterns after oxytocin compared to L-DOPA administration highlight a divergent role of each neurochemical in modulating the neural mechanisms underlying social performance monitoring.

**Supplementary Information:**

The online version contains supplementary material available at 10.1007/s00213-024-06541-9.

## Introduction

Behaving in an adaptive and flexible manner requires individuals to monitor their own actions or performance continuously, such that they can detect deviations from their goals and adjust their behavior accordingly. Performance monitoring is therefore an essential component of adaptive and goal-directed behavior. Functional magnetic resonance imaging (fMRI) studies have highlighted that performance-monitoring processes involve several emotional and cognitive brain regions. While correct responses and positive outcomes have been associated with ventral striatum (VS) activity, erroneous responses and negative performance feedback typically activate the posterior medial frontal cortex (pMFC) and the anterior insula (AI) (de Bruijn et al. 2009; Koban et al. 2013; Krönke et al. 2018; Overgaauw, Jansen, & de Bruijn 2020; Radke et al. 2011a).

The neurotransmitter dopamine is thought to play a key role in performance monitoring (Holroyd & Coles 2002; Ullsperger et al. 2014), which is based on the finding that midbrain dopamine neurons firing rates increase when outcomes are better than expected (positive prediction errors or PEs) and decrease when outcomes are worse than expected (negative PEs) (Schultz 2022). Dopamine involvement in the computation of PEs is supported by research indicating that increasing dopamine using the precursor L-DOPA increases BOLD responses to (positive) reward PEs in the striatum (e.g., Pessiglione et al. 2006). Additionally, there is an abundance of electrophysiological evidence for a key role of dopamine in performance monitoring, with pharmacological studies showing that administration of dopamine agonists increases error-related brain activity, as reflected in amplitudes of the so-called error-related negativity (ERN) (Barnes et al. 2014; De Bruijn et al. 2004, 2005; Spronk et al. 2016), while dopamine antagonists decrease ERNs (De Bruijn et al. 2006; Zirnheld et al. 2004).

Most studies investigating modulatory effects on performance monitoring have focused solely on individual contexts, where errors only affect oneself. However, as social beings, our actions and specifically our mistakes often also affect the people around us. Importantly, monitoring potential consequences of our own actions for others facilitates optimizing their outcomes and enables prosocial behavior (Carlo 2013). Studies have shown that the social context may modulate activity in performance-monitoring regions. For example, activity in pMFC and AI is enhanced when errors result in painful versus non-painful outcomes for a friend (Koban et al. 2013) and when participants are fully responsible for painful outcomes compared to when they share responsibility for errors with another recipient (Cui et al. 2015). Recent studies from our lab indicate increased ERNs when errors have negative consequences for others (de Bruijn et al. 2020) and suggest that the extent to which the social context affects performance-monitoring activity depends on individual differences such as psychopathic- (Overgaauw et al. 2020) and obsessive–compulsive traits (Jansen & de Bruijn 2020).

Interestingly, dopamine may also play an important role in social performance monitoring. Upregulating dopamine may have domain-general effects through a direct impact on dopamine-driven PEs, thus affecting performance monitoring regardless of whether participants act for themselves or others. Indirect support for this comes from work in rodents showing that social and non-social PEs activate the same dopamine-innervated regions (Manduca et al. 2021) and that dopamine neurons also code social PEs (Solié et al. 2022). However, it has also been shown that administering dopamine precursor L-DOPA in an economic bargaining game reduced hyperaltruistic tendencies of preferring harming oneself over harming others (Crockett et al. 2015) and increased selfish behavior in healthy men (Pedroni et al. 2014). Additionally, blocking dopamine transmission using amisulpride during an interpersonal decision task reduced prosocial behavior in women and selfish behavior in men (Soutschek et al. 2017), reduced the perceived self-relevance of others’ actions (Barnby et al. 2023) and led to increased reciprocal behavior in a repeated trust game (Mikus et al. 2023). Upregulating dopamine might thus also lead to a self-serving bias where personal outcomes become more salient than those of others, at least in men.

Another neurochemical relevant for social performance monitoring is the neuropeptide oxytocin. While oxytocin was initially suggested to facilitate prosocial behavior (e.g., MacDonald & MacDonald 2010), this view has been challenged with recent studies emphasizing the context and baseline dependency of such effects (Harari-Dahan & Bernstein 2014; Quintana & Guastella 2020; Shamay-Tsoory & Abu-Akel 2016). The social salience hypothesis proposes that rather than universally enhancing prosocial behaviors, oxytocin increases the salience of social cues (Shamay-Tsoory & Abu-Akel 2016). In line with this, a recent study (Martins et al. 2022) found dose-dependent modulations of oxytocin on neural PE encoding specifically during prosocial learning. Note that the different nasal administration method used by Martins et al. (2022) prevents direct comparability with studies using standard nasal devices due to potential differences in terms of the effective central concentrations of oxytocin reached. We recently found that a 24 international units (IU)-dose of oxytocin led to opposing tracking of prosocial versus self-benefitting PEs in several brain regions including the pMFC and insula, such that there was negative signaling in the prosocial context and positive signaling when performing for oneself after placebo, whereas signaling was less negative or positive in the prosocial context and negative when performing for oneself after oxytocin. Exploratory analyses also showed that more positive encoding of prosocial PEs in these regions after oxytocin were related to higher learning rates in the prosocial context (Jansen et al. 2022). In addition, we demonstrated oxytocin involvement in social performance monitoring, with oxytocin-induced enhancements in ERN amplitudes specifically for social mistakes (de Bruijn et al. 2017). These findings suggest that oxytocin may particularly facilitate (pro)social performance monitoring.

Importantly, the social salience theory also proposes that these oxytocin effects are achieved via interactions with the brain’s dopaminergic system (Shamay-Tsoory & Abu-Akel 2016). In support of this notion, there is for example evidence from preclinical studies that oxytocin stimulation facilitates dopamine release during social reward (Hung et al. 2017). There is also indirect evidence from human studies suggesting that oxytocin can enhance (reward-related) brain activity in dopamine-dependent brain areas (e.g., Mickey et al. 2016; Scheele et al. 2013). Yet, human studies addressing whether dopamine and oxytocin can produce convergent regional effects are missing. Hence, in this study we manipulated drugs acting on each neurochemical in one single study, allowing us to establish their potential neural overlap and differences.

Here we pharmacologically manipulated dopamine and oxytocin in men to investigate the neurochemical mechanisms underlying performance monitoring of own actions when one is responsible for own versus others’ outcomes. Investigating the neural mechanisms underlying (pro)social performance monitoring is essential, given that functional alterations in performance monitoring, (pro)social behavior and dopaminergic and oxytocinergic systems are implicated in a wide range of clinical conditions (see e.g., Diederen & Fletcher 2021; Koo et al. 2010; Lutz et al. 2021a, b; Lutz et al. 2021a, b; Riesel et al. 2019; Willemssen et al. 2008). Studying these mechanisms may thus help us gain a better understanding of these conditions. Using a double blind, cross-over design, participants performed the Cannonball task, a computerized shooting game (de Bruijn et al. 2009; Overgaauw et al. 2020; Radke et al. 2011a). Mistakes either affected the participants’ own monetary bonus or the bonus of an anonymous other participant. We hypothesized that L-DOPA would either enhance activation in performance-monitoring regions (pMFC, AI and VS) in a self-serving or domain-general manner and that oxytocin would specifically enhance activity in these same regions in the social context, when mistakes affect others. We additionally conducted whole-brain analyses to investigate whether social context and drugs recruit and/or modulate brain regions involved in (pro)social behavior outside the performance-monitoring network (Koban & Pourtois 2014; Radke et al. 2011a).

## Method

### Participants

Based on medium effect sizes (Cohen's f = 0.25) found in previous pharmacological studies (e.g., Soutschek et al. 2017; Vo et al. 2018; Vo et al. 2016), a priori calculations indicated a required sample size of *N* = 26 (assuming a correlation between repeated measures of 0.5 and a power of 85%). To account for drop-out (10%), we recruited 30 healthy participants (aged 18–35, *M* = 22.8, *SD* = 3.6) with good command of the Dutch language. Only males were included in the study in order to avoid menstrual cycle-dependent interactions (Jocham et al. 2011, 2014). Exclusion criteria included: cardiovascular, endocrine, psychiatric, neurological or hematological disease, MRI counter-indications, drug/medication history in preceding month, participation in other drug study in preceding 3 months, > 3 units of alcohol/day, > 5 cigarettes/day, and > 8 on the anxiety/depression subscale of the Hospital Anxiety and Depression Scale (Pouwer et al. 1997). Participants were asked to abstain from caffeine, alcohol, and smoking 24 h prior and to not eat/drink (except water) 1.5 h prior to lab arrival. Our study was approved by the medical ethical committee of the Leiden University Medical Centre and registered at the Dutch CCMO Register (NL68645.058.19). Participants provided written informed consent and received €140 upon completion.

### Procedure

Participants were recruited using social media including Facebook and the Leiden university Leiden University Research Participation System (SONA). After expressing their willingness to participate, they received an information letter via email and filled out an online screening questionnaire in Qualtrics. If they met the inclusion criteria, a telephone appointment was made to assess contraindications for the MRI scanner and to arrange lab sessions. Participants visited the lab three times, with a minimum interval of 7 days (mean- / mode- / maximum interval = 13.6 / 7 / 46 days) between visits to ensure drug washout (Jocham et al. 2011, 2014). The sessions always took place between 9:00 AM and 5:30 PM, with a maximum within-person time difference between sessions of 1.5 h.

**Fig. 1:**
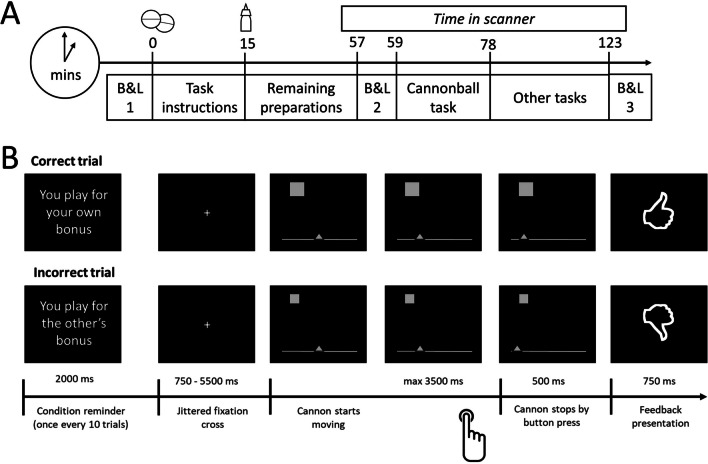
Overview of the timetable for each session (**A**) and the Cannonball task (**B**). (**A**) During each session participants received a pill followed by a nasal spray exactly 15 min later (either a placebo pill followed by a placebo spray, a placebo pill followed by a spray containing oxytocin, or a L-DOPA pill followed by placebo spray). Participants performed the Cannonball task in the scanner approximately 59 min (*SD* = 2.5) after pill administration and 44 min (*SD* = 3.3) after administration of the nasal spray. Subjective drug effects (alertness, mood and anxiety) were assessed using the Bond and Lader (1974) mood rating scale (B&L) right before administration of the pill (T1), approximately one hour later (T2), and at the end of the visit (T3). (**B**) The goal of the Cannonball task is to stop a horizontally moving cannon (triangle) by a button press, to precisely line it up with a stationary target (square). Participants played this task in two conditions: for their own monetary bonus and for the bonus of another participant, whereby errors resulted in a monetary deduction from an initial bonus. At the beginning of each trial a black screen with a white fixation cross was presented, the duration of which was jittered using a randomized jitter list with the following durations in ms: 750, 750, 1000, 1000, 1000, 1250, 1250, 1500, 1500, 5500. Subsequently, the target and cannon appeared on the screen. Target location was randomly determined on each trial, whereas the cannon was always horizontally centered. Immediately after presentation, the cannon starts moving either to the right or the left for a maximum of 3500 ms or until a button is pressed. In case of a missed response, the words ‘TOO LATE’ appeared on the screen. 500 ms after a response was made, an unambiguous feedback signal (thumb up/thumb down) was presented for 750 ms, indicating whether the response was correct or incorrect. Each run lasted 80 trials, and after every 10 trials participants were reminded of the recipient condition (“You are playing for the bonus of the other participant” vs “You are playing for your own bonus”). The task was presented using E-prime 3.0 software (Psychology Software Tools, Pittsburgh, PA)

In a cross-over, double-blind design, participants received either L-DOPA (100 mg levodopa with 25 mg carbidopa) or a placebo pill, followed by either oxytocin (24 IU / 0.37 ml per nostril) or a placebo (chlorobutanol) nasal spray using a double-dummy technique (see timetable in Fig. 1A). Placebo and L-DOPA pill were repacked in identical Swedish orange capsules to keep the study double-blind. Previous studies have shown that the selected dosage of L-DOPA reach maximum plasma concentrations after approximately 50 min, with an elimination half-life of 80–90 min (Nyholm et al. 2012). For oxytocin, the evidence for the impact of intranasal oxytocin administration on central levels of oxytocin is primarily indirect or from animal studies (Quintana et al. 2021). Whereas some studies have suggested that effects of oxytocin can be observed as early as 20–60 min after administration, other research suggests that a 24-IU dose of oxytocin may take up to 75 min to increase concentrations of oxytocin in cerebrospinal fluid (Striepens et al. 2013). Some human work measuring resting regional cerebral blood flow observed OT-induced changes over a 25–78 min interval with a peak response at 39–51 min (Paloyelis et al. 2016) and highlights the importance of considering the specific method of intranasal administration (Martins et al. 2020). Importantly, a recent study similarly using a standard nasal spray with a 24 IU dose found the most robust effects on amygdala reactivity between 45–70 min after administering oxytocin (Spengler et al. 2017). Hence, we aimed to start within this timeframe. By administering the nasal spray exactly 15 min after the pill, we therefore ensure that the task commenced at peak concentrations for both drug.

To assess subjective effects of the drugs, the Bond-Lader mood rating scale was administered (Bond & Lader 1974, reported elsewhere at: https://doi.org/10.31234/osf.io/h7yrz) at three different timepoints (see Fig. 1) and participants answered questions about their subjective responses to the task after each condition of the task and at the end of every session (Table S4). Respiration and heart rate during scanning were continuously monitored using a breathing belt and finger clip (peripheral pulse unit).

### Experimental task

Participants performed a variant of the Cannonball Task in the scanner (Fig. 1B; de Bruijn et al. 2009; Overgaauw et al. 2020; Radke et al. 2011b). The aim of the task is to stop a horizontally moving cannon (triangle) by a button press using the index finger of their dominant hand, precisely lining it up with a stationary target to shoot the target (square). Participants responded using fiber-optic buttons that were attached to their legs. To ensure comparable error rates between participants, the size of the target was dynamically adapted based on participant’s performance, such that a mean hit rate of 63% was achieved. This was done to maintain a similar number of errors and correct trials for each individual, which allowed for comparisons across drugs and social context. The target size adjustment was calculated after each trial by comparing the current success rate to the desired success rate using the following formula: Change = ((Actual_Percentage_Correct -Goal_Percentage_Correct)* *Change_*Factor) / (100* Current_Target Size). The Change_Factor was set at 0.25 in line with previous studies using the same paradigm (see de Bruijn et al. 2009; Overgaauw et al. 2020; Radke et al. 2011b). If the current percentage was higher than the goal percentage, the change was subtracted from the current target size. If the current percentage was lower than the goal percentage, the change was added. This dynamic process was implemented anew at the beginning of each condition.

Participants performed the task in two different conditions, the order of which was counterbalanced between participants. In one condition, they played for their own monetary bonus, whereas in the other condition, their errors negatively affected the bonus of another participant. They were additionally informed that the other participant would be randomly chosen and would remain anonymous, and that this participant in his turn played for someone else’s bonus, in order to prevent feelings of reciprocity. Importantly, none of the participants reported disbelief in the task at debriefing. In each recipient condition participants started with a bonus of €5, and for every mistake 10 cents were deducted. Correct responses had no effect on the bonus (cf. de Bruijn et al. 2009). For ethical reasons, participants received a fixed €10 bonus following the final visit.

### Behavioral analysis

Data analyses were performed in Rstudio version 1.3.959 (R Core Team 2020). To confirm that our dynamical adaptation of target sizes (see section Experimental task above) was successful in achieving a comparable error rate across participants, we analyzed accuracy rates as a manipulation check. Because target sizes increase when participants commit a lot of errors and decrease when participants make too little errors, the size of these targets was used as a measure of task performance, whereby small target sizes represent better performance. Accuracy rates and target sizes were analyzed with a logistic generalized linear mixed model (GLMMs) and a linear mixed model (LMM), respectively, on the full trial-by-trial dataset using the lme4 package (Bates et al. 2014). Analysis of the accuracy rates included correctness as binary outcome variable and fixed effects for drug and recipient. The target size analysis contained fixed effects of drug, recipient, and correctness. Post hoc comparisons were carried out using the emmeans package (Lenth et al. 2018) and corrected for multiple testing using a false discovery rate (FDR) at *p* < 0.05 (Benjamini & Hochberg 1995). All models contained random intercepts for participants to account for dependency in the data. The random-effects structure for each LMM was determined according to the procedure described in (Bates et al. 2015), which consists of 1) fitting the maximal random-effects structure or, if the maximal model does not converge or is degenerate, fitting a reduced zero-correlation parameter model, 2) removing random effects estimated at zero or close to zero that do not result in a significant loss of goodness of fit according to a likelihood-ratio test, and 3) extending the model with correlation parameters again (only) if this improves model fit. The final random-effects structure of each model can be found in the analysis script on https://osf.io/5jhvk/.

To quantify the evidence for or against the effects of task performance, we additionally computed Bayes factors (BFs) using a Bayesian repeated measures (rm) ANOVA (JASP Team 2020) with default priors. BFs represents the probability ratio of observed data under one model versus another and thus provides an index of the relative strength of evidence for the null (BF_01_) or alternative (BF_10_) hypothesis (Marsman & Wagenmakers 2017). A BF_10_ > 1 and < 1 favors the alternative hypothesis and null hypothesis, respectively. BFs between 0.33–3 are considered anecdotal evidence indicating data insensitivity. BFs between 3–10, 10–30 and 30–100 or between 0.33–0.1, 0.1–0.03, and 0.03–0.01 are considered moderate, strong and very strong evidence for the alternative or null hypothesis, respectively (Quintana & Williams 2018). For significant interactions we report the matched models BF inclusion (BF_incl_) (Clyde et al. 2011), which computes the evidence for a particular factor by comparing all models with a particular factor to equivalent models without that factor.

### fMRI data acquisition, preprocessing and analysis

MRI data was acquired at the LUMC using a 3.0 T Philips scanner with a 32-channel head coil. 3DT1 structural images were acquired using 155 slices (FOV 195.8 × 250 × 170.5 mm, voxel size = 1.1 mm^3^, 0 mm slice gap, matrix size = 228 × 177, flip angle = 8°) with a TR of 7.9 ms and a TE of 3.5 ms, resulting in a total scan duration of 4:11 min. Functional scans were acquired in two separate runs using 40 transverse slices in descending order (FOV 220 × 220 × 120.7, matrix size = 80 × 77, voxel size = 2.75 mm^3^, slice gap = 0.275 mm, flip angle = 80°) with a TR of 2200 ms and a TE of 30 ms. The first two volumes of each run were discarded to allow for equilibration of T1 saturation effects. Duration of each run was approximately 7–8 min, depending on reaction time during the task. Head motion was restricted using foam inserts.

Imaging data were preprocessed and analyzed using SPM12 (Wellcome Trust Centre for Neuroimaging, University College London). Preprocessing was performed for each session separately and consisted of the following steps: slice-time correction, correction for field-strength inhomogeneity’s using b0 field maps, unwarping and realignment, coregistration to subject-specific structural images, segmentation, normalization to MNI space using the DARTEL toolbox (Ashburner 2007) and smoothing using an 8-mm full width half maximum isotropic Gaussian kernel.

On the first level, we defined a general linear model for each session for each participant with separate regressors indicating the (zero duration) feedback onsets to correct and incorrect responses in each condition (placebo_self_correct, placebo_self_error, placebo_other_correct, placebo_other_error / L-DOPA_self_correct, L-DOPA_self_error, L-DOPA_other_correct, L-DOPA_other_error / oxytocin_self_correct, oxytocin_self_error, oxytocin_other_correct, oxytocin_other_error). Furthermore, we included regressors for stimulus and text onsets for each run and modelled these for their specific durations. In case of missed responses, a separate regressor for missed trials was included as well. The stimulus onset was parametrically modulated by target size to capture variance related to differences in size of the target across trials. To capture residual effects of head motion, we additionally included the 6 realignment parameters for each run and included censor regressors (Siegel et al. 2014) for volumes with more than 1 mm scan-to-scan motion or more than 4 mm absolute motion. First-level contrast maps for each condition were submitted to a second-level flexible factorial model with 12 levels, where we computed main and interaction effects for drug, recipient and correctness using t-contrasts.

We created a single region of interest (ROI) mask (see Fig. 4A) by combining two anatomical masks of the bilateral VS (Harvard–Oxford Atlas) and the AI (Neuromorphometrics, Inc. as provided in SPM12) with a 10-mm radius sphere of the pMFC [x = 4, y = 32, z = 38], based on the peak coordinates of the error versus correct contrast from a previous study using the same paradigm (de Bruijn et al. 2009). Effects are reported at *p* < 0.05 family-wise error (FWE) corrected at the voxel level and effects in ROIs at *p* < 0.05 FWE-small volume corrected (SVC), applying an extent-threshold of *k* = 5. Paired t-tests were used to test extracted cluster estimates to understand the direction of interaction effects.

## Results

### Behavioral data

#### Manipulation check

The GLMM of the accuracy rates confirmed that our adaptive criterion was successful in obtaining a stable error rate across conditions, as there were no significant effects of drug, recipient or their interaction (all *t*s < 0.69, all *p*s > 0.49, with a mean accuracy rate of 63–64% in each drug-by-recipient condition).

#### Task performance

**Fig. 2:**
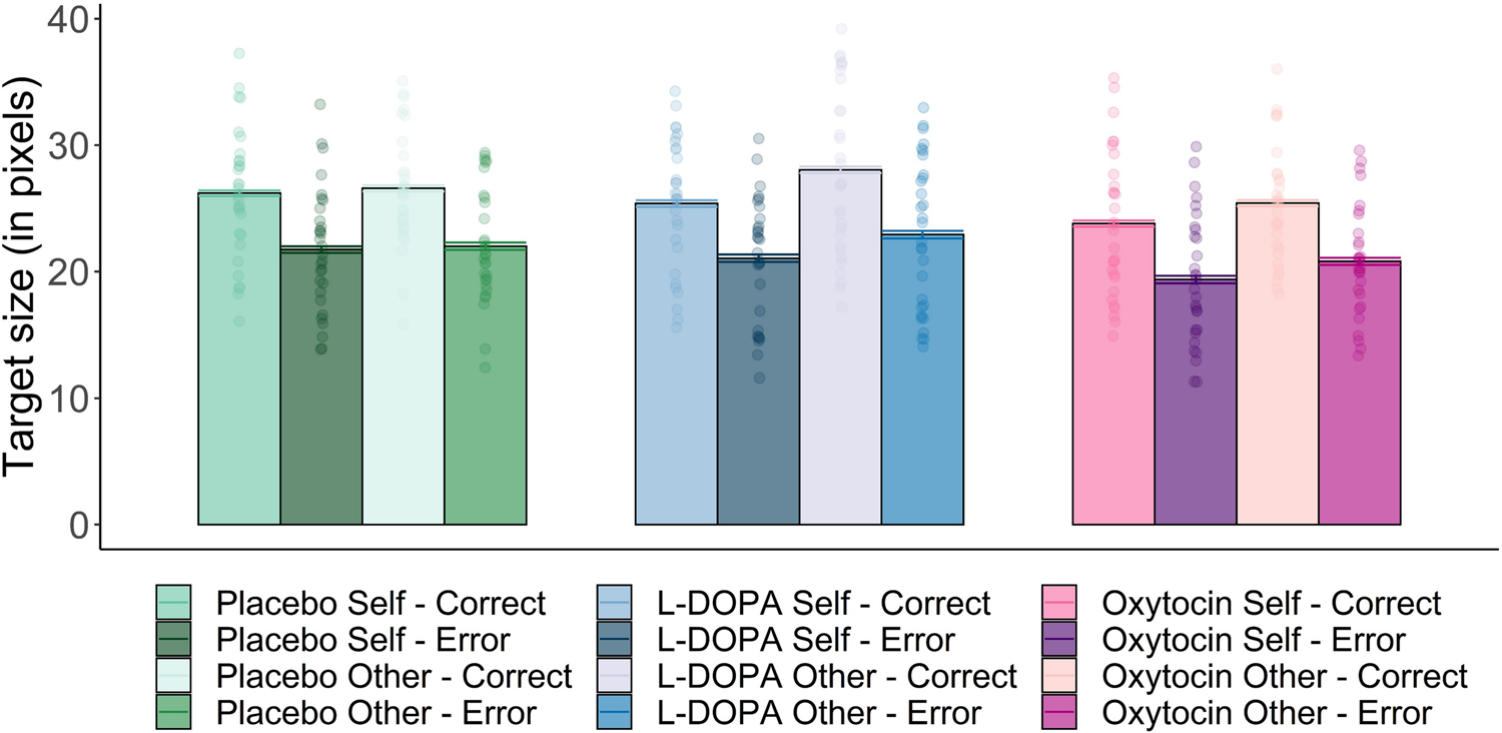
Mean target size (in pixels) for correct and incorrect trials across drug and recipient conditions. As accuracy rate was kept constant by reducing or increasing the target sizes, smaller target sizes reflect better performance. The Fig. demonstrates the main effects of correctness (smaller target sizes for errors), recipient (smaller target sizes for self) and drug (smaller target sizes after oxytocin). Error bars represents standard error of the mean

Target size was analyzed as a measure of task performance (Fig. 2). The LMM showed the expected main effect of correctness (*b* = -4.5, *SE* = 0.1, *t* = -33.20, *p* < 0.001), with larger target sizes for correct responses (*M* = 25.9, *SE* = 0.7) compared to errors (*M* = 21.3, *SE* = 0.7). Additionally, target sizes were significantly smaller after oxytocin (*M* = 22.3, *SE* = 0.8) compared to placebo (*M* = 24.1, *SE* = 0.7; *b* = -1.8, *SE* = 0.6, *t* = -2.78, *p* = 0.009) and L-DOPA (*M* = 24.3, *SE* = 0.9; *b* = -2.0, *SE* = 0.8, *Z* = 2.40, *p* = 0.020). Furthermore, a significant effect of recipient (*b* = 1.4, *SE* = 0.6, *t* = 2.44, *p* = 0.021) indicated smaller target sizes when participants performed for their own bonus (*M* = 22.9, *SE* = 0.7) compared to when they played for other’s bonus (*M* = 24.3, *SE* = 0.7), independent of the drug condition. Notably, this recipient effect seemed to be primarily driven by L-DOPA (self vs other: *b* = -2.27, *SE* = 1.02, *Z* = -2.23, *p* = 0.030), and oxytocin (self vs other: *b* = -1.52, *SE* = 0.79, *Z* = -1.93, *p* = 0.050), but not by the placebo condition (self vs other: *b* = -0.36, *SE* = 1.03, *Z* = -0.35, *p* = 0.73). There was no significant main effect of L-DOPA (*b* = 1.89, *SE* = 0.77, *t* = 0.25, *p* = 0.81), nor any significant interactions between recipient and L-DOPA (*b* = 1.92, *SE* = 1.35, *t* = 1.42, *p* = 0.17). Oxytocin did also not interact with recipient (*b* = 1.16, *SE* = 1.22, *t* = 0.96, *p* = 0.35).

A Bayesian rm ANOVA including the same factors revealed extremely strong evidence for the main effect of correctness (BF_incl_ = 1.02e + 22) and drug (BF_incl_ = 227.20). Bayesian post hoc tests showed extremely strong evidence for a difference between oxytocin and placebo (BF_10_ = 138.63) and strong evidence for a difference between oxytocin and L-DOPA (BF_10_ = 22.03), but moderate evidence against a difference between L-DOPA and placebo (BF_10_ = 0.11). Strong evidence was also found for the main effect of recipient (BF_incl_ = 29.05), while the evidence against an interaction between drug and recipient was anecdotal (BF_incl_ = 0.32).

### fMRI data

#### Both own and other’s errors activate expected performance monitoring regions after placebo

**Fig. 3:**
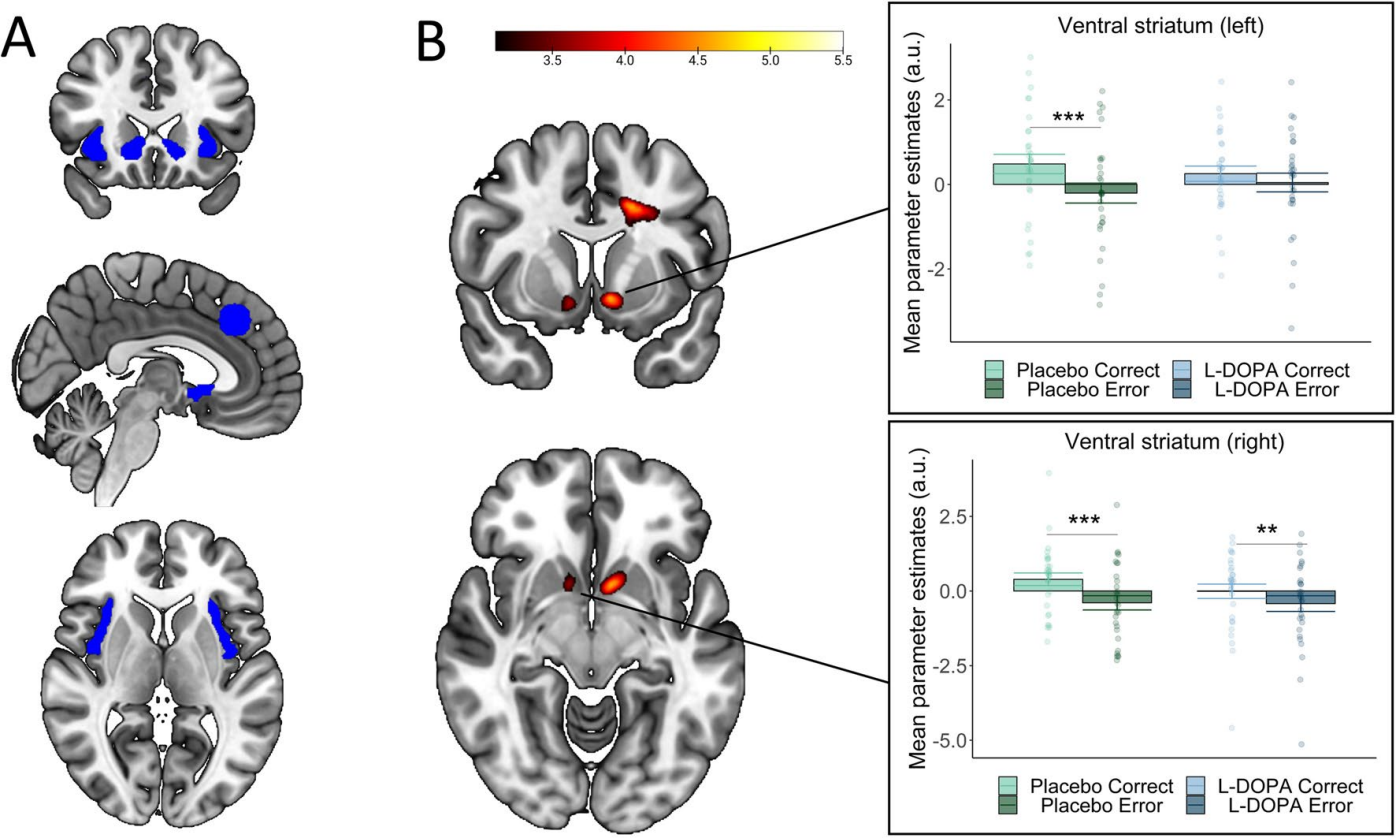
Region-of-interest (ROI) mask (**A**) and interactions between L-DOPA and correctness in the left (**B**) and right (C) ventral striatum. (A) We created a single ROI mask by combining anatomical masks of the ventral striatum and anterior insula with a 10-mm sphere of the posterior medial prefrontal cortex [x = 4, y = 32, z = 38]. (B & C) Whole-brain image and extracted parameter estimates of the significant interaction between L-DOPA and correctness in the left and right ventral striatum (left: x = -9, y = 9, z = -8], *k* = 89, *Z* = 4.61, *p* = 0.001 SVC-FWE; right: x = 11, y = 8, z = -11, *k* = 7, Z = 3.65, *p* = 0.043 SVC-FWE). Contrasts are displayed at *P* < 0.001 for illustration purposes [x = -9, y = 9, z = -8]. *p < 0.05, **p < 0.01

We first confirmed that expected performance-monitoring regions were activated in the placebo condition. Whole-brain contrasts showed the expected regions, with activation in pMFC and AI for the Error > Correct contrast and in the VS for the Correct > Error contrast, *p*s < 0.001 FWE. Conjunction analyses additionally showed that errors versus correct responses activated the pMFC and bilateral insula both when playing for one’s own bonus and when playing for the other participant’s bonus (all *p*s < 0.001 FWE, Fig. S1). Similarly, left and right VS were activated in both recipient conditions for correct responses compared to errors, *p*s < 0.002 FWE. Region of interest (ROI) analyses (Fig. 3A) for the placebo condition only revealed no main effects of recipient nor any interactions between recipient and correctness. Table S2 provides a complete list of significant whole-brain clusters.

#### Reduced error-correct differentiation in ventral striatum after L-DOPA versus placebo.

Next, we tested for drug effects and interactions with recipient within our ROIs. We observed an interaction between correctness and drug (L-DOPA vs placebo) in the left and right VS (left: x = -9, y = 9, z = -8], *k* = 89, *Z* = 4.61, *p* = 0.001 SVC-FWE; right: [x = 11, y = 8, z = -11], *k* = 7, *Z* = 3.65, *p* = 0.043 SVC-FWE, Fig. 3B). Inspection of the parameter estimates indicates that both interactions are driven by that fact that there is attenuated differentiation between error- and correct-related activation after L-DOPA (left VS: *p* = 0.11; right VS: *p* = 0.005) compared to placebo (*p*s < 0.001). Whole brain results are presented in Table S3.

#### Enhanced activity in pregenual anterior cingulate cortex when errors affect others after oxytocin

**Fig. 4:**
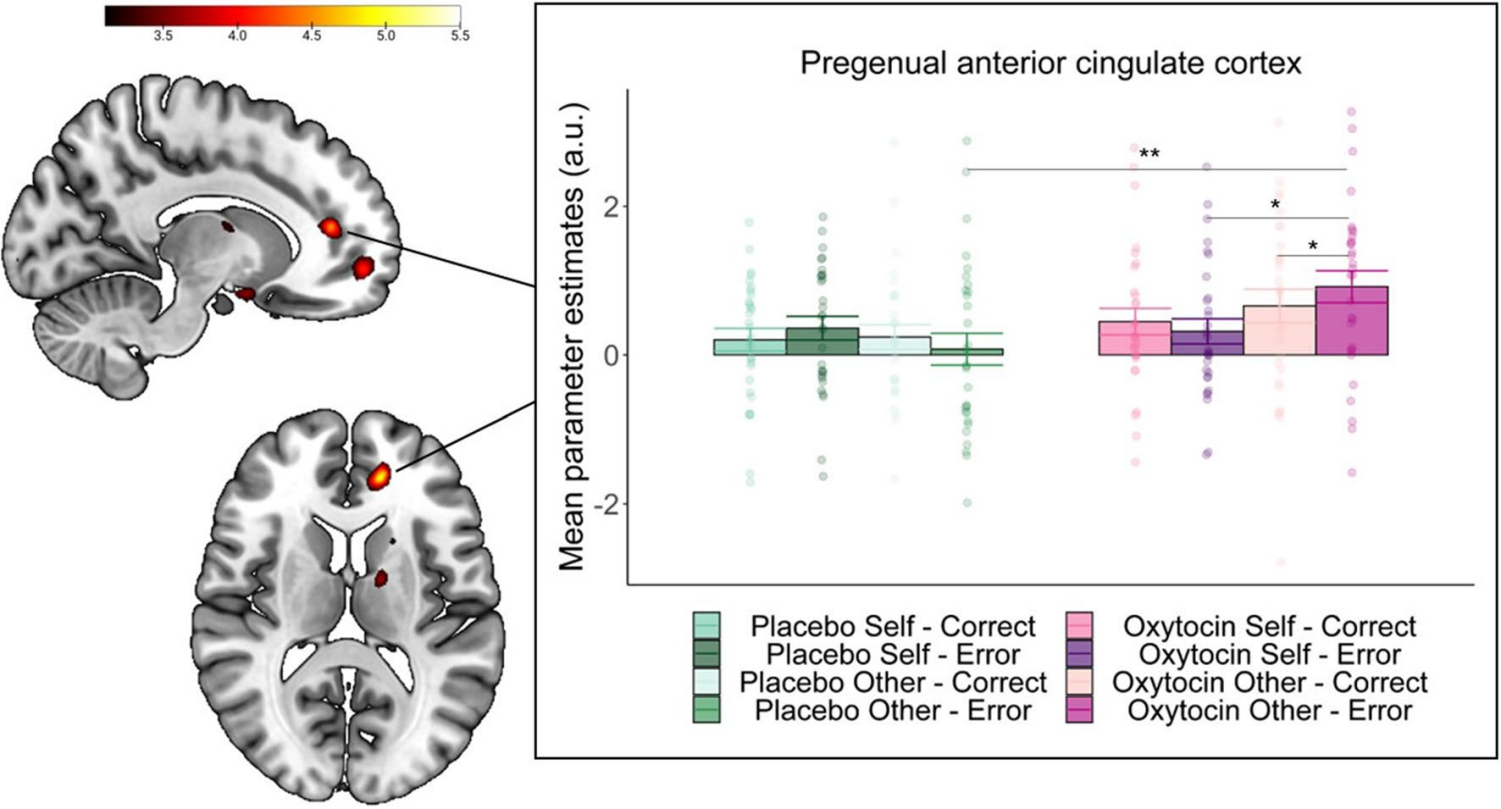
Whole-brain image and extracted parameter estimates for the interaction between oxytocin, recipient and correctness in the left pregenual anterior cingulate cortex. (A) Whole-brain image and extracted parameter estimates show enhanced activation for errors that affect the other after oxytocin (x = -15, y = 42, z = 12, *k* = 12, *Z* = 5.00, *p* = 0.007 FWE). Contrasts are displayed at *p* < 0.001 for illustration purposes [x = -12, y = 42, z = 12]. *p < 0.05, **p < 0.01

We observed no significant drug effects of oxytocin nor any interactions with recipient within our ROIs. Interestingly, however, exploratory whole brain analysis did reveal a significant interaction between drug (oxytocin vs placebo), recipient and correctness in a cluster within the pregenual anterior cingulate cortex (pgACC; x = -15, y = 42, z = 12, *k* = 12, *Z* = 5.00, *p* = 0.007 FWE). Inspection of the parameter estimates (Fig. 4) for this region indicates that activity in this region was significantly enhanced for errors that affected others after oxytocin compared to errors that affected others after placebo (*p* = 0.002), as well as compared to errors that affected the self after oxytocin (*p* = 0.017) and compared to correct responses that affected others after oxytocin (*p* = 0.024). The same interaction contrast additionally revealed a small cluster in the precentral gyrus (Table S3).

#### Associations of neural modulations with target size and self-reported motivation

We performed some exploratory analyses to test whether drug-induced modulations of neural activity was correlated with behavioral performance or motivation. To this end, we applied LMMs to the extracted parameter estimates for each drug effect with target size or self-reported motivation as mean-centered continuous predictors. Details on self-reported state outcomes are provided in supplemental material and Table S4.

For the VS, we found main effects of self-reported motivation in both the left and right VS (left VS: *b* = -0.30, *SE* = 0.14, *t* = -2.17, *p* = 0.034; right VS: *b* = -0.36, *SE* = 0.16, *t* = -2.28, *p* = 0.027), but no interaction of motivation with L-DOPA or correctness (*t*s < 1.56, *p*s > 0.12), indicating that higher motivation predicted lower parameter estimates in these regions, independent of the drug condition and of whether responses were correct or incorrect. No main effects of- or interactions with target sizes were found (*t*s < 0.46, *p*s > 0.65).

**Fig. 5:**
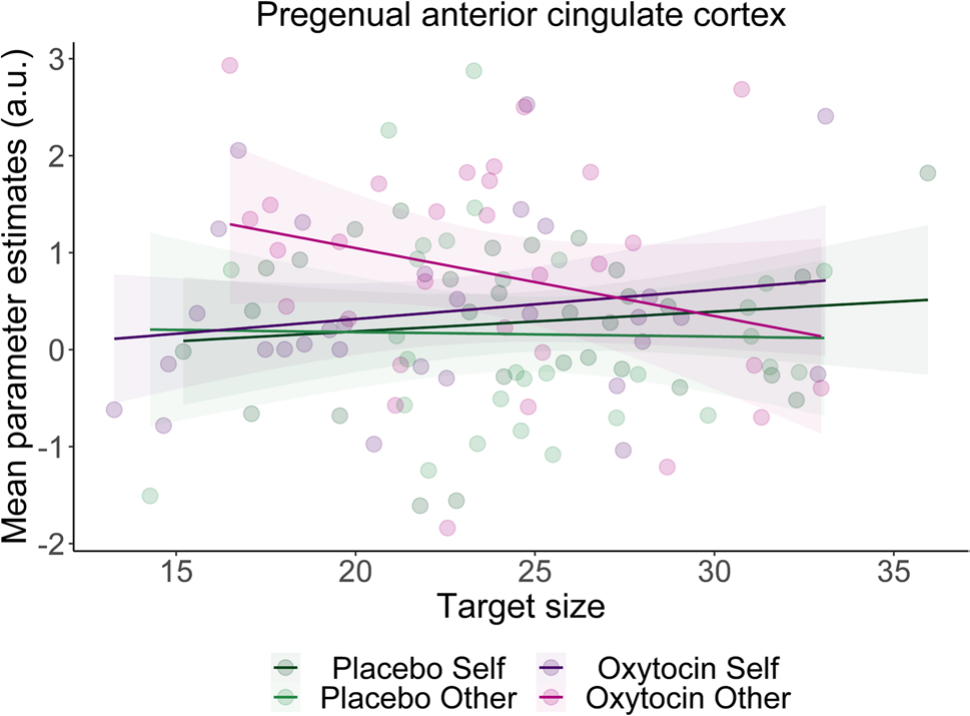
Scatterplot depicting the association between target size and mean parameter estimates in the pregenual anterior cingulate cortex after oxytocin and placebo per recipient condition. Plot shows that better performance (as indicated by lower target sizes) is associated with higher activity in the pregenual anterior cingulate cortex specifically when playing for others after oxytocin

For the pgACC, the LMM revealed a significant interaction between oxytocin, recipient and target size (*b* = 0.77, *SE* = 0.28, *t* = 2.76, *p* = 0.008). Further inspection of this interaction indicated that independent of whether responses were correct or incorrect, smaller target sizes (i.e., better performance) predicted higher activity in this region specifically when playing for others after oxytocin (*b* = -0.51, *SE* = 0.21, 95% CI [-0.93, -0.09]), with this slope differing significantly from outcomes that affected self after oxytocin (*b* = -0.67, *SE* = 0.23, *t* = -2.85, *p* = 0.01) and also showing a trend-level difference with outcomes affecting others after placebo (*b* = 0.52, *SE* = 0.28,* t* = 1.85, *p* = 0.07). No main effects of- or interactions with motivation were found (*t*s < 1.65, *p*s > 0.10). Figure 5 shows the relation between target sizes and parameter estimates in the pgACC for each recipient and drug condition.

## Discussion

The aim of the current study was to improve our understanding of the role of the neurochemicals dopamine and oxytocin in individual and social performance monitoring, by pharmacologically manipulating both chemicals in a single study. After placebo, the expected involvement of performance-monitoring regions (posterior medial frontal cortex [pMFC], anterior insula [AI], ventral striatum [VS]) was found when comparing processing of correct with incorrect responses. Moreover, our conjunction analysis showed that these regions were involved in both recipient conditions. This highlights a domain-general function of these regions in performance monitoring regardless of whether one is responsible for their own or someone else’s outcome, though it should be acknowledged that a distinction between social or domain-general processes may still be present within the same voxels through different multivariate patterns, or involve distinct algorithm or computational levels (see, e.g., Lockwood et al. 2020). Moreover, we found no significant differences in neural activation between playing for oneself versus another after placebo. Behaviorally, we did observe a main effect of recipient, indicating improved performance when playing for oneself versus the anonymous other. However, it is worth noting that this difference was not significant when considering the placebo condition separately and that differences in size of the target did not result in actual differences in monetary outcomes between recipient conditions, since the accuracy rate was kept under experimental control. We did not observe differences in self-reported states such as motivation between the two recipient conditions either. This suggests that participants did not make a clear distinction in terms of motivational- or affective significance between playing for oneself or another after placebo. fMRI results did indicate an interaction between L-DOPA and correctness in the bilateral VS demonstrating reduced differentiation between errors and correct responses after L-DOPA compared to placebo, independent of recipient. Furthermore, while oxytocin did not modulate activity within our ROIs, the whole-brain analysis indicated an interaction between oxytocin, correctness and recipient in the pregenual anterior cingulate cortex (pgACC), with specifically enhanced activity in this region after oxytocin for errors that affected the other participant. On a behavioral level, we additionally found that participants performed significantly better, as indicated by smaller target sizes, under influence of oxytocin compared to both placebo and L-DOPA, independent of the recipient condition. Exploratory analyses additionally indicated that lower target size predicted higher activity in the pgACC specifically when playing for others after oxytocin.

In contrast to our hypothesis, we found that -rather than increasing- L-DOPA reduced the difference between correct- and error-related activation within specific clusters in the bilateral VS, suggesting attenuated performance-monitoring activity in this region, independent of the recipient condition. Furthermore, we did not find support for L-DOPA to impact performance-monitoring activity in pMFC or AI, nor did it significantly alter behavioral performance. Given that enhanced ERNs are linked to increased BOLD activity in performance-monitoring regions (Ullsperger et al. 2014), the current findings appear in contrast with EEG evidence for enhanced performance monitoring after administration of dopamine agonists. It should however be noted that electrophysiological changes take place on a much smaller timescale than the BOLD signal in fMRI studies and used other dopamine agonists with different pharmacokinetic profiles (Martins et al. 2017), making direct comparison with our findings difficult.

Our findings contrast with an fMRI study (Pessiglione et al. 2006) where enhanced PE signaling in the VS after L-DOPA administration was reported. However, it is important to consider that this study used a between-subject design with a small sample size (*N* = 13 per group) and the results only showed an increase in signaling compared to the dopamine antagonist haloperidol and not compared to placebo. The study also included both male and female participants, unlike our exclusively male sample. This may be relevant given previous work indicating that males have greater dopamine release in the VS compared to females (Munro et al. 2006). Another study reporting increased VS signaling after L-DOPA focused specifically on older adults, who are more likely to show suboptimal dopamine functioning than our sample of healthy young adults (Chowdhury et al. 2013). In line with our current findings, we recently found – using a social probabilistic learning paradigm in the same sample—that L-DOPA blunted the signaling of positive PEs in the VS, also independent of whether performance affected the outcomes of another person or oneself (Jansen et al. 2023). Two other studies using different types of dopamine agonists, namely methamphetamine (Bernacer et al. 2013) and methylphenidate (Evers et al. 2017) also report reduced PE signaling in the VS. The authors argued that this was due to methylphenidate’s increase in tonic dopamine levels, which may lead to a reduced rather than enhanced impact of (phasic) PEs through increased stimulation of presynaptic dopamine autoreceptors (Beaulieu & Gainetdinov 2011) and which may reduce postsynaptic responses to phasic dopamine (Jonasson et al. 2014). This may be relevant to the current findings, as L-DOPA also increases tonic levels of dopamine (Harun et al. 2016). This idea is also congruent with the dopamine overdose hypothesis, which states that in healthy individuals, dopamine agonists may impair adequate functioning of the VS due to an overdosing of this already optimally-functioning brain area (Cools 2006). In line with this notion, recent work shows that methylphenidate was found to boost striatal prediction error signaling in healthy individuals with low baseline dopamine tone (synthesis capacity), but reduced it in those with high baseline dopamine tone (van den Bosch et al. 2022). Together, these studies substantiate the hypothesis that L-DOPA might have blunted error-related brain activity in the VS by reducing the dynamic range for phasic prediction errors.

It should be acknowledged that unlike previous fMRI investigations of PEs, our task did not involve a learning component and the contrast between correct responses and errors is likely to also involve the more hedonic impact of receiving positive versus negative feedback. Regarding this more hedonic aspect, our results align with a recent study in which L-DOPA was found to attenuate the VS response to reward in a two-stage Markov decision-task (Kroemer et al. 2019), though this effect did not survive multiple comparison correction. Notably, a recent review highlights the mixed findings of fMRI studies on both PE signals and more hedonic processing of rewards in the brain following L-DOPA as well as other dopamine agonists (Webber et al. 2021).

Importantly, the effect of L-DOPA on VS activity was recipient-independent, suggesting that upregulating dopamine impacts the neural mechanisms underlying performance monitoring regardless of whether actions affect oneself or someone else. We explicitly focused on a social context in which outcomes for others did not impact one’s own outcomes, while previous studies suggesting self-serving biases after L-DOPA employed tasks that involved a trade-off where others’ rewards came at personal costs (Crockett et al. 2015; Pedroni et al. 2014; Soutschek et al. 2017). This may explain the contrasting outcomes. Notably, we did observe a somewhat larger self-bias in performance under influence of L-DOPA, though there was no significant interaction between recipient and L-DOPA. Hence, it will be important to explore different types of social performance-monitoring paradigms in future studies, with for example situations where benefitting others requires personal costs or where a reciprocity component is involved. Additionally, our results are not generalizable to female samples. Given past indications of gender differences in L-DOPA's effects on social decision-making (Soutschek et al. 2017), further research on sex and gender effects is important.

We hypothesized that effects of oxytocin would interact with recipient, such that oxytocin would specifically facilitate performance monitoring when playing for the other participant. While we did not observe oxytocin-induced modulations within our predefined performance-monitoring regions, our whole-brain analyses revealed an interaction with recipient and correctness in the pgACC. Specifically, error-related activation was enhanced following oxytocin when playing for the other. Interestingly, this region, which shows strong functional connectivity with performance-monitoring regions (Palomero-Gallagher et al. 2019), resembles a cluster found in a previous study from our lab (Radke et al. 2011a). There, the area was more active for erroneous and correct outcomes that additionally had monetary consequences for another participant compared to outcomes that only affected oneself. Moreover, the cluster in the current study includes the gyrus part of the ACC (ACCg), a region which has been specifically linked to the processing of social information (see e.g., Lockwood et al. 2020). For example, using neurophysiological recordings in monkeys it was demonstrated that neurons in the ACCg preferentially encoded rewards delivered to another monkey (Chang et al. 2013). Single unit recordings in humans have shown that neurons in the ACCg signal PEs when monitoring others’ outcomes (Hill et al. 2016). Additionally, studies have demonstrated that the ACCg is activated when processing cues that are predictive of others’ reward (Apps et al. 2016; Lockwood et al. 2015), and encodes PEs while learning about ownership for other’s but not own outcomes (Lockwood et al. 2018). Moreover, the region has been proposed to encode motivational salience, as activity in the ACCg has been found to increase similarly in response to negative affective cues as to rewarding stimuli (Apps et al. 2016). This may explain why the current results show activity in this region to be especially pronounced during error trials, which signal the greatest motivational salience in terms of required adjustments. Further, a recent study showed that this area encodes representations of prosocial effort, and scaled with how much effort was required when making choices for others, suggesting an important role for the ACCg in motivating actions that benefit others (Lockwood et al. 2022). This also fits with the observation of the current study that increased activity after oxytocin in this region when playing for others, was related to improved performance. This suggests an oxytocin-induced role of this region in implementing efforts when actions affect others, though it should be noted that this correlation is exploratory and should be interpreted with cautioun given previous indications that reproducible brain-wide association studies require thousands of participants (Marek et al. 2022). Moreover, these analyses were conducted using neural signals at the mean-level as outcome. Future research should aim to model these neural signals on a single-trial level as well, to further strengthen the conclusion that this region is associated with behavioral performance.

Interestingly, we observed recipient-independent behavioral improvements in performance after oxytocin. This finding is not easily explained by the social salience hypothesis (Shamay-Tsoory & Abu-Akel 2016) and seems more in line with recent theories highlighting the non-social influences of oxytocin such as the allostatic theory (Quintana & Guastella 2020). According to this theory, the primary role of oxytocin is to facilitate stability in changing environments, which it does by acting on physiological and psychological systems that mediate how we sense and react to changes and that support learning and prediction of future events. From this perspective, the general improvement of performance after oxytocin could be achieved via improved adaptation to the dynamically changing target cue. Moreover, the observed recruitment of the pgACC in the social condition could speculatively reflect enhanced processing or efforts of socially salient information to facilitate behavioral performance in the social context in order to reach a similar level of performance improvement as during the self-benefitting context.

We did not observe any communalities in neural activity after L-DOPA and oxytocin. Our current findings thus hint at a divergent roles of these pharmacological substances in modulating the neural correlates of performance monitoring. Given these distinct neural patterns, the socially-specific neural modulations that we observed after oxytocin could potentially be explained by direct (salience) effects on oxytocin receptors present in the pregenual ACC (Quintana et al. 2019) rather than a more indirect modulation of dopaminergic pathways. However, it is also important to recognize that neural effects of oxytocin could still be achieved via the dopaminergic system, as oxytocin can regulate dopamine signaling and might determine when, how much and where in the brain this neurotransmitter is released (Quintana et al. 2019; Shamay-Tsoory & Abu-Akel 2016). Hence, the fact that we do not observe the same neural activity patterns after oxytocin compared to L-DOPA does not provide evidence for the notion that effects of oxytocin are not achieved via dopaminergic modulations. It would be important for future research examining regional overlap of dopamine and oxytocin to consider different types and dosages of each drug, and take into account differences in timing and baseline dopamine levels (see e.g., Webber et al. 2021). Additionally, to truly disentangle possible interactions between dopamine and oxytocin, future research should aim to manipulate both systems at the same time (e.g., administering oxytocin together with a dopamine antagonist).

Note that we limited our ROI analyses to the pMFC, AI and VS based on previous evidence for recipient- and drug-dependent modulations of these regions. However, there are several other regions that may be involved in (social) performance monitoring. For example, the habenular complex has been implicated in the processing of negative feedback during performance monitoring (Ullsperger & Von Cramon 2003) and in the processing of reward prediction errors (see e.g., Baker et al. 2016). Hence, the role of this brain area may be relevant to investigate in future research.

## Conclusion

In summary, we observed reduced error-correct differentiation in the VS after L-DOPA versus placebo. Whereas this effect of L-DOPA was domain-general, after oxytocin both domain-general and recipient-specific drug effects were observed. Analyses revealed an interaction of oxytocin with recipient in the pgACC, showing specifically enhanced activity in this region after oxytocin for errors that affected the other. This shows that oxytocin plays a role in neural responses to errors that affect other people's outcomes, by specifically recruiting the pgACC when processing socially salient information. Behaviorally, our results showed a general improvement in performance after oxytocin. Additionally, increased pgACC activity in the social context after oxytocin was related to smaller target sizes, suggesting that oxytocin-induced recruitment of this area may contribute to improved behavioral performance. Importantly, we observed different behavioral and neural patterns after oxytocin compared to L-DOPA administration, highlighting their distinct role in modulating the mechanisms underlying performance monitoring.

### Supplementary Information

Below is the link to the electronic supplementary material.

Supplementary file1 (DOCX 245 KB)
