# Supplementary material for: L-DOPA and oxytocin influence the neural correlates of performance monitoring for self and others
Source: Psychopharmacology (Berl). 2024 Jan 29;241(5):1079–92. doi: 10.1007/s00213-024-06541-9 (PMC11031497; doi:10.1007/s00213-024-06541-9)
Supplement: Supplementary file 1 — Supplementary file1 (DOCX 245 KB) [file 213_2024_6541_MOESM1_ESM.docx]

Supplementary material for

L-DOPA and oxytocin influence the neural correlates of performance monitoring for self and others

Myrthe Jansen ^1,2^, Sandy Overgaauw ^1,2^ and Ellen R. A. de Bruijn^1,2^

^1^Institute of Psychology, Leiden University, The Netherlands

^2^Leiden Institute for Brain and Cognition (LIBC), Leiden, The Netherlands

* Correspondence concerning this article should be addressed to:

Myrthe Jansen, Department of Clinical Psychology, Leiden University, Wassenaarseweg 52, 2333 AK Leiden, The Netherlands, Phone: +31 71 527 2727. Email: [m.jansen@fsw.leidenuniv.nl](mailto:m.jansen@fsw.leidenuniv.nl).

**SUPPLEMENTARY RESULTS**

**Behavioral data (first versus second half of the task)** As an exploratory analysis, we reanalyzed the behavioral data using the first and second half of the task separately (trials 1-40 vs trials 41-80).

Similar to the analyses of the full trial data, there were no significant effects of drug, recipient or their interaction on the accuracy rates for neither the first (*p*s > .36) or second (*p*s > .20) half of the trials.

Next, we conducted analyses of the target sizes for the first versus second half of the task. Means and standard deviations for each half in each drug by recipient condition are presented in Table S1. Both the first and second half showed the expected effects of correctness (*p*s < .001) with larger target sizes for correct responses compared to errors. Additionally, in the first half, target sizes were significantly smaller after oxytocin (*M* = 23.7, *SE* = .96) compared to placebo (*M* = 26.0, *SE* = .77; *b* = -2.3, *SE* = 1.0, *t* = -2.3, *p* = .029), in line with the full trial data. The difference in target size between oxytocin and L-DOPA (*M* = 25.9, *SE* = .96) was however not significant here (*b* = 2.2, *SE* = 1.4, *z* = 1.5, *p* = .13). In the second half, the main effect of oxytocin was not significant (oxytocin vs placebo: *t* = -1.69, *p* = .102). In contrast, the main effect of recipient was present in the second half (*b* = 2.0, *SE* = 0.7, *t* = 2.66, *p* = .013). but not in the first half of the task (*t* = 1.05, *p* = .3). Furthermore, in the first half a marginal interaction emerged between L-DOPA and correctness (*b* = -0.9, *SE* = 0.5, *t* = -1.96, *p* = .051). However, there were no significant post-hoc comparisons. No other significant effects were observed for either the first (*t*s < 1.44, *p*s > .15) nor second half of the task (*t*s < 1.52, *p*s > .14).

**Table S1.** Means and standard deviations for target sizes (as an index of task performance) for the first (trial 1-40) versus second half (trial 41-60) of the task for each drug by recipient condition.

|  |  |  | First half (trial 1-40) | | Second half (trial 41-80) | |
| --- | --- | --- | --- | --- | --- | --- |
| Drug | Recipient | Correctness | *M* | *SD* | *M* | *SD* |
| Placebo | Self | Correct | 28 | 9.0 | 24 | 8.2 |
|  |  | Error | 24 | 8.0 | 20 | 7.1 |
|  | Other | Correct | 28 | 9.2 | 25 | 9.3 |
|  |  | Error | 24 | 8.6 | 20 | 8.5 |
| L-DOPA | Self | Correct | 28 | 10.4 | 23 | 9.0 |
|  |  | Error | 23 | 9.6 | 19 | 7.8 |
|  | Other | Correct | 29 | 9.9 | 27 | 10.4 |
|  |  | Error | 24 | 8.6 | 22 | 9.2 |
| Oxytocin | Self | Correct | 25 | 10.0 | 22 | 9.6 |
|  |  | Error | 21 | 9.1 | 18 | 8.1 |
|  | Other | Correct | 27 | 10.6 | 24 | 8.3 |
|  |  | Error | 22 | 9.4 | 20 | 7.4 |

**fMRI data**


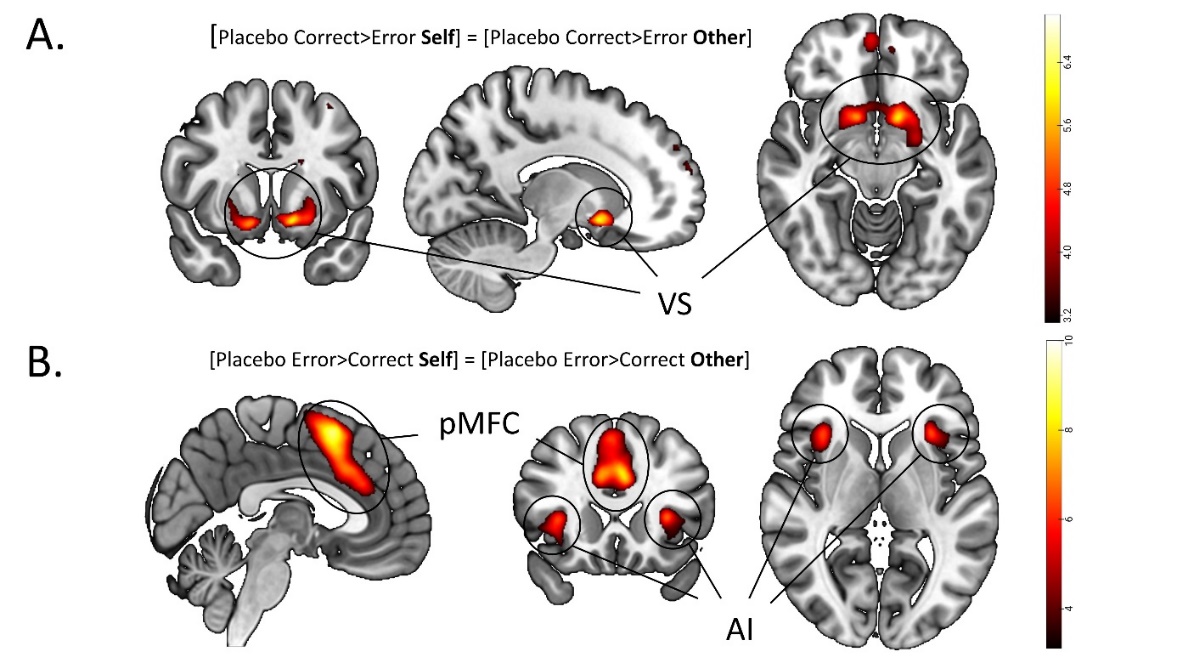


**Figure S1.** Whole brain contrasts for the Self-Other conjunction after placebo. (A) Whole-brain conjunction for Correct > Error shows activation of the ventral striatum (VS) both when playing for oneself and the other participant. x = -14; y = 9; z = -11. (B) Whole brain conjunction for Error > Correct shows activation of the posterior medial prefrontal cortex (pMFC) and anterior insula (AI) both when playing for oneself and the other participant. X = 3; y = 21; z = 5. Contrasts are displayed at *p* < .001 for illustration purposes.

**Table S2**. Overview of whole brain effects of recipient and correctness for the placebo condition only.

| Brain region | L/R | x | y | z | k | t | z |
| --- | --- | --- | --- | --- | --- | --- | --- |
| **Self > Other** |  |  |  |  |  |  |  |
| No suprathreshold voxels |  |  |  |  |  |  |  |
| **Other > Self** |  |  |  |  |  |  |  |
| No suprathreshold voxels |  |  |  |  |  |  |  |
| **Correct > Error** |  |  |  |  |  |  |  |
| Ventral striatum | R | 15 | 9 | -12 | 3859 | 9.30 | Inf |
| Ventral striatum | L | -12 | 11 | -11 |  | 8.68 | Inf |
| Putamen | L | -24 | 3 | -6 |  | 7.69 | 7.38 |
| Superior frontal gyrus,  dorsolateral | R | 27 | 26 | 56 | 607 | 7.80 | 7.48 |
| Superior frontal gyrus,  dorsolateral | R | 24 | 36 | 50 |  | 6.17 | 6.01 |
| Middle frontal gyrus | R | 38 | 17 | 56 |  | 4.98 | 4.89 |
| Superior frontal gyrus,  dorsolateral | L | -20 | 33 | 50 | 1715 | 7.43 | 7.15 |
| Middle frontal gyrus | L | -21 | 26 | 54 |  | 7.13 | 6.88 |
| Middle frontal gyrus | L | -33 | 15 | 56 |  | 6.35 | 6.17 |
| Superior frontal gyrus,  medial orbital | R | 5 | 59 | -8 | 922 | 6.75 | 6.54 |
| Superior frontal gyrus,  medial orbital | L | -11 | 51 | -9 |  | 5.75 | 5.62 |
| Crus I of cerebellar hemisphere | R | 14 | -84 | -32 | 436 | 6.49 | 6.30 |
| Crus I of cerebellar hemisphere | R | 38 | -75 | -32 |  | 6.03 | 5.88 |
| Crus I of cerebellar hemisphere | R | 29 | -80 | -29 |  | 5.54 | 5.42 |
| Middle occipital gyrus | L | -14 | -102 | 3 | 158 | 6.01 | 5.86 |
| Superior occipital gyrus | L | -8 | -101 | 9 |  | 5.68 | 5.55 |
| Supplementary motor area | R | 6 | -26 | 56 | 344 | 5.82 | 5.69 |
| Paracentral lobule | L | -5 | -27 | 56 |  | 5.58 | 5.45 |
| Caudate | R | 20 | 12 | 18 | 90 | 5.53 | 5.41 |
| Caudate | R | 21 | 2 | 20 |  | 4.70 | 4.63 |
| Inferior occipital gyrus | L | -29 | -96 | -11 | 79 | 5.35 | 5.25 |
| Cuneus | R | 18 | -95 | 9 | 93 | 5.34 | 5.23 |
| Caudate | L | -18 | 8 | 26 | 55 | 5.03 | 4.94 |
| Middle cingulate & paracingulate  gyri | R | 5 | -53 | 32 | 173 | 4.99 | 4.90 |
| Middle cingulate & paracingulate  Gyri | L/R | 0 | -44 | 33 |  | 4.94 | 4.86 |
| Superior frontal gyrus,  Dorsolateral | R | 17 | 66 | 17 | 5 | 4.75 | 4.67 |
| **Error > Correct** |  |  |  |  |  |  |  |
| Supplementary motor area | R | 3 | 12 | 56 | 7572 | 13.49 | Inf |
| Middle cingulate & paracingulate  gyri | L | -5 | 21 | 33 |  | 11.25 | Inf |
| Middle cingulate & paracingulate  gyri | R | 8 | 24 | 32 |  | 10.85 | Inf |
| Insula | R | 35 | 21 | 5 | 1246 | 9.42 | Inf |
| Inferior frontal gyrus,  opercular part | R | 57 | 14 | -2 |  | 4.72 | 4.64 |
| Insula | L | -32 | 21 | 6 | 1103 | 9.19 | Inf |
| SupraMarginal gyrus | L | -63 | -42 | 32 | 324 | 6.05 | 5.89 |
| SupraMarginal gyrus | R | 66 | -29 | 30 | 122 | 5.78 | 5.64 |
| Middle frontal gyrus | L | -29 | 50 | 15 | 84 | 5.54 | 5.42 |
| Middle frontal gyrus | R | 32 | 48 | 30 | 169 | 5.51 | 5.39 |
| Middle frontal gyrus | L | -30 | 41 | 26 | 88 | 5.12 | 5.02 |
| Precuneus | R | 14 | -71 | 62 | 12 | 4.74 | 4.66 |
| Calcarine | L | -15 | -68 | 6 | 5 | 4.73 | 4.65 |
| **[Self – Error > Correct] =**  **[Other – Error > Correct]** |  |  |  |  |  |  |  |
| Supplementary motor area | R | 3 | 12 | 56 | 3971 | 9.28 | Inf |
| Middle cingulate & paracingulate  gyri | L | -5 | 21 | 33 |  | 8.28 | Inf |
| Middle cingulate & paracingulate  gyri | R | 8 | 24 | 32 |  | 7.72 | 7.41 |
| Insula | L | -32 | 21 | 6 | 190 | 6.85 | 6.63 |
| Insula | R | 35 | 21 | 5 | 356 | 6.49 | 6.30 |
| **[Self – Correct > Error] =**  **[Other –Correct > Error]** |  |  |  |  |  |  |  |
| Putamen | L | -14 | 9 | -11 | 381 | 6.19 | 6.02 |
| Putamen | L | -26 | 3 | -5 |  | 5.25 | 5.14 |
| Ventral striatum / Nucleus accumbens | R | 14 | 11 | -12 | 84 | 5.54 | 5.42 |
| Superior frontal gyrus,  dorsolateral | R | 26 | 24 | 57 | 39 | 5.32 | 5.22 |
| Superior frontal gyrus,  medial orbital (vmPFC) | R | 5 | 57 | -9 | 46 | 4.98 | 4.89 |
| **Correctness * recipient - Correct:-Self[-1]-Other[1], Error:-Self[1]-Other[-1]** |  |  |  |  |  |  |  |
| No suprathreshold voxels |  |  |  |  |  |  |  |
| **Correctness * recipient - Correct:-Self[1]-Other[-1], Error:-Self[-1]-Other[1]** |  |  |  |  |  |  |  |
| No suprathreshold voxels |  |  |  |  |  |  |  |

*Note.* Regions are labelled according to the Automated anatomical labelling atlas 3. Results are corrected for multiple comparisons using a voxel-level family-wise error (FWE) correction at P<0.05. Extent-threshold *k* = 5.

**Table S3**. Whole-brain effects of drug and interactions between drug, recipient and correctness.

| Brain region | L/R | x | y | z | k | t | z |
| --- | --- | --- | --- | --- | --- | --- | --- |
| **Placebo > L-DOPA** |  |  |  |  |  |  |  |
| No suprathreshold voxels |  |  |  |  |  |  |  |
| **L-DOPA > Placebo** |  |  |  |  |  |  |  |
| Fusiform gyrus | R | 41 | -42 | -12 | 58 | 5.32 | 5.21 |
| **Placebo > Oxytocin** |  |  |  |  |  |  |  |
| No suprathreshold voxels |  |  |  |  |  |  |  |
| **Oxytocin > Placebo** |  |  |  |  |  |  |  |
| No suprathreshold voxels |  |  |  |  |  |  |  |
| **L-DOPA * correctness – Placebo:-Correct[1]-Error[-1], L-DOPA:-Correct[-1]-Error[1]** |  |  |  |  |  |  |  |
| Crus I of cerebellar hemisphere | R | 15 | -77 | -32 | 134 | 5.34 | 5.23 |
| Crus I of cerebellar hemisphere | R | 38 | -72 | -30 | 29 | 5.10 | 5.00 |
| **L-DOPA * correctness – Placebo:-Correct[-1]-Error[1], L-DOPA:-Correct[1]-Error[-1]** |  |  |  |  |  |  |  |
| No suprathreshold voxels |  |  |  |  |  |  |  |
| \| **Oxytocin * correctness – Placebo:-Correct[-1]-Error[1], L-OT:-Correct[1]-Error[-1]** \| \| --- \| \| No suprathreshold voxels \| |  |  |  |  |  |  |  |
| \| **Oxytocin * correctness – Placebo:-Correct[-1]-Error[1], OT:-Correct[1]-Error[-1]** \| \| --- \| \| No suprathreshold voxels \| |  |  |  |  |  |  |  |
| **L-DOPA * correctness * recipient – Placebo Correct:-Self[1]-Other[-1], Placebo Error:-Self[-1]-Other[1], L-DOPA Correct:-Self[-1]-Other[1], L-DOPA Error:-Self[1]-Other[-1]** |  |  |  |  |  |  |  |
| No suprathreshold voxels |  |  |  |  |  |  |  |
| **Oxytocin * correctness * recipient – Placebo Correct:-Self[1]-Other[-1], Placebo Error:-Self[-1]-Other[1], L-OT Correct:-Self[-1]-Other[1], L-DOPA Error:-Self[1]-Other[-1]** |  |  |  |  |  |  |  |
| No suprathreshold voxels |  |  |  |  |  |  |  |
| **Oxytocin * correctness * recipient – - Placebo Correct:-Self[-1]-Other[1], Placebo Error:-Self[1]-Other[-1], OT Correct:-Self[1]-Other[-1], L-DOPA Error:-Self[-1]-Other[1]** |  |  |  |  |  |  |  |
| Pregenual ACC | L | -15 | 42 | 12 | 12 | 5.10 | 5.00 |
| Precentral gyrus | R | 39 | -12 | 57 | 6 | 4.77 | 4.69 |
| *Note*. Regions are labelled according to the Automated anatomical labelling atlas 3. Results are corrected for multiple comparisons using a voxel-level family-wise error (FWE) correction at *p* < .05. Extent-threshold *k* = 5. | | | | | | | |

**Self-reported states** After completing each condition of the Cannonball task (questions 1-7) and at the end of every session (questions 8-13), participants were asked to indicate to what extent they agreed with a number of statements about the task using Likert scales (see Table S4 for questions and mean scores). Results from logistic ordinal regression analyses (polr from the MASS package; Venables & Ripley, 2013) showed no significant differences in self-reported states between playing for oneself versus another participant (*p*s > .18). Besides trend-level effects showing that participants disliked making mistakes less after L-DOPA compared to placebo (*b* = -.58, *SE* = .33, *t* = -1.74, *p* = .081), and that participants perceived the task as less difficult after oxytocin compared to placebo (*b* = -.84, *SE* = .48, *t* = -1.75, *p* = .080), there were no significant effects of drugs (*p*s > .13) nor any interactions between recipient and drugs (*p*s > .46).

We did not find any subjective effects (mood, anxiety, alertness) of either drugs (reported elsewhere at <https://doi.org/10.31234/osf.io/h7yrz>).

**Table S4.** Means and standard deviations for self-reported states.

|  |  | Placebo | | L-DOPA | | Oxytocin | |
| --- | --- | --- | --- | --- | --- | --- | --- |
| *Question* |  | *M* | *SD* | *M* | *SD* | *M* | *SD* |
| 1. I was afraid to make mistakes | *for the other* | 2.7 | 1.3 | 2.9 | 1.5 | 2.8 | 1.5 |
|  | *for myself* | 2.9 | 1.6 | 2.6 | 1.3 | 2.6 | 1.5 |
| 2. I disliked making mistakes | *for the other* | 4.3 | 1.9 | 3.6 | 1.7 | 4.1 | 1.6 |
|  | *for myself* | 4.1 | 1.9 | 3.7 | 1.7 | 4.1 | 1.6 |
| 3. I felt frustrated when making a mistake | *for the other* | 4.2 | 1.7 | 3.9 | 1.7 | 4.0 | 1.9 |
|  | *for myself* | 4.4 | 2.1 | 3.8 | 1.7 | 4.4 | 1.8 |
| 4. I felt guilty when making mistakes | *for the other* | 2.9 | 1.6 | 3.1 | 1.4 | 3.0 | 1.8 |
|  | *for myself* | 2.6 | 1.6 | 3.0 | 1.5 | 3.0 | 1.7 |
| 5. I liked performing well | *for the other* | 5.4 | 1.4 | 5.7 | 1.3 | 5.9 | 1.0 |
|  | *for myself* | 5.7 | 1.4 | 5.6 | 1.4 | 5.9 | 1.2 |
| 6. I was motivated to perform well | *for the other* | 5.7 | 1.4 | 5.4 | 1.1 | 5.7 | 1.3 |
|  | *for myself* | 5.8 | 1.3 | 5.7 | 1.5 | 6.0 | 1.2 |
| 7. I felt responsible for my mistakes | *for the other* | 4.3 | 1.8 | 4.2 | 1.7 | 4.1 | 1.9 |
|  | *for myself* | 4.6 | 1.8 | 4.3 | 1.9 | 4.2 | 1.7 |
| 8. I thought the game was (1) very annoying to (7) very nice  9. I thought the game was (1) very easy to (7) very difficult | | 5.1 | 1.3 | 4.8 | 1.1 | 5.0 | 1.3 |
|  |  | 3.9 | 0.8 | 3.5 | 0.9 | 3.5 | 1.0 |
| 10. Awareness of monetary losses (1- not at all, 7- very) | | 4.2 | 1.6 | 4.3 | 1.8 | 4.4 | 1.8 |
| 11. Disliked mistakes the most for 1) self – 7) other | | 3.3 | 1.4 | 2.9 | 1.2 | 3.2 | 1.5 |
| 12. Connected to other (1- not at all, 7- very connected) | | 3.3 | 1.6 | 3.0 | 1.6 | 3.3 | 1.5 |
| 13. Concentration (1 – not at all, 7 – very concentrated) | | 5.1 | 1.4 | 4.8 | 1.4 | 5.4 | 1.2 |

*Note*. Unless indicated otherwise, statements were answered on a scale ranging from 1 (strongly disagree) to 7 (strongly agree).

**Drug allocation guess** Fisher’s exacts test indicated that participants did not guess their drug condition above chance level (*p* = .1), see Table S5.

**Table S5.** Actual drug condition (rows) versus drug guess (columns).

|  | Placebo | L-DOPA | Oxytocin |
| --- | --- | --- | --- |
| Placebo | 15 | 8 | 6 |
| L-DOPA | 7 | 10 | 12 |
| Oxytocin | 12 | 12 | 5 |

*Note*. Data is missing for one participant in the L-DOPA and Oxytocin condition and for one participant in the Placebo condition (these participants circled two options instead of one).

**Associations between target size modulations and self-reported motivation.** We also explored whether the observed target size modulations would be correlated with changes in self-reported motivation (“I was motivated to perform well”). However, adding self-reported motivation as a mean-centered continuous predictor to the target size LMM revealed no significant main effects of- or interactions with motivation (all *t*s > 1.14, *p*s > .257).

**SUPPLEMENTARY REFERENCES**

Venables, W. N., & Ripley, B. D. (2013). *Modern applied statistics with S-PLUS*: Springer Science & Business Media.
